# Gut and skin microbiome-metabolome pathways in vitiligo: from dysbiosis to immune activation and melanocyte dysfunction

**DOI:** 10.3389/fimmu.2026.1952635

**Published:** 2026-09-15

**Authors:** Viviana A. Ruiz-Pozo, Rafael Tamayo-Trujillo, Andrés S. Cadena-Paredes, Patricia Guevara-Ramírez, Santiago Cadena-Ullauri, Elius Paz-Cruz, Ana Karina Zambrano

**Affiliations:** Centro de Investigación Genética y Genómica, Facultad de Ciencias de la Salud Eugenio Espejo, Universidad UTE, Quito, Ecuador

**Keywords:** autoimmune inflammation, dysbiosis, gut–skin axis, melanocyte dysfunction, metabolome, microbiome, vitiligo

## Abstract

Vitiligo is an autoimmune depigmenting disorder driven by genetic susceptibility, oxidative stress, immune dysregulation, and progressive melanocyte loss. Emerging microbiome–metabolome evidence suggests that gut and skin dysbiosis may contribute to disease progression through the gut–skin axis. This review integrates current evidence linking microbial imbalance with immune activation and melanocyte dysfunction in vitiligo. Studies in patients and experimental vitiligo models show altered gut and skin microbial diversity, enrichment of inflammation-associated taxa, depletion of commensal bacteria, impaired short-chain fatty acid biosynthesis, and disruption of tryptophan–indole, kynurenine, bile acid, taurine, riboflavin, and oxidative stress-related metabolic pathways. These alterations may weaken epithelial barrier integrity, impair immune tolerance, promote systemic low-grade inflammation, and increase melanocyte vulnerability to mitochondrial dysfunction, reactive oxygen species accumulation, impaired melanogenesis, apoptosis, and autoantigen exposure. Dysbiosis-associated metabolic remodeling may affect key immunometabolic pathways, including AhR-mediated tryptophan–kynurenine signaling, SCFA-dependent GPCR/HDAC regulation, IFN-γ–JAK/STAT–CXCL9/CXCL10 inflammatory amplification, and NF-κB/inflammasome-mediated innate immune priming. Together, these mechanisms may enhance dendritic cell activation, Th1/Th17 polarization, autoreactive CD8+ T-cell recruitment, and persistence of melanocyte-specific resident memory T cells. Current evidence supports a biologically plausible link between microbiome–metabolome disruption and autoimmune melanocyte loss in vitiligo. However, causality remains unproven. Longitudinal, multi-omics, and functional studies are required to define whether microbial and metabolic signatures act as biomarkers, mechanistic drivers, or therapeutic targets in vitiligo.

## 1 Introduction

Vitiligo is an autoimmune skin disorder characterized by the progressive loss of melanocytes, leading to depigmented macules that tend to enlarge over time (1, 2). In the general population, the prevalence of vitiligo is estimated at 0.36%, although this figure rises to 0.67% among adults, who account for the majority of cases (3, 4). However, data from the Global Vitiligo Atlas (GLOVA) suggest a higher global prevalence of 1–2%, although this figure remains approximate, largely owing to underreporting and limited surveillance in regions such as Africa, South America, and rural Asia (5). The risk architecture of vitiligo is predominantly genetic, with heritable factors accounting for an estimated 80% of the attributable risk-ranging from common polymorphisms to rare variants-whereas environmental exposures contribute the remaining 20% (6).

Oxidative stress plays a key role in the pathogenesis of vitiligo. This imbalance between the production of reactive oxygen species (ROS) and the availability of antioxidants is particularly relevant in melanocytes, given that melanogenesis itself generates high levels of ROS (7, 8).

Emerging evidence suggests that the gut microbiota may also contribute to the multifactorial etiology of vitiligo, although its precise role remains incompletely understood. Dysbiosis, defined as an imbalance in the microbial community, may disrupt intestinal homeostasis and exert downstream effects on the skin. Proposed mechanisms include alterations in the production of short-chain fatty acids (SCFAs), which are essential for metabolic regulation; changes in tryptophan metabolism, with a shift of the kynurenine pathway (KYN) toward the overproduction of pro-inflammatory metabolites and a reduction in the serotonergic (5-HT) and indole pathways, which are normally associated with anti-inflammatory molecules capable of protecting melanocytes; as well as changes in the production of secondary bile acids by gut bacteria, which may compromise their protective role against oxidative stress and increase melanocyte vulnerability (2, 9). In addition, some studies have attributed more specific functions to microbiota, such as the production of ROS or secondary metabolites, including hippuric acid, which can activate enzymes such as NOS2 and MAPK14 and, at high concentrations, induce oxidative stress and damage melanocytes (10).

In the present article, we review current microbiome, metabolome, and pathway-level evidence linking gut and skin dysbiosis with immune activation, oxidative stress, and melanocyte dysfunction in vitiligo. We focus on how dysbiosis-associated metabolic remodeling may influence host–microbe communication and activate interconnected molecular pathways involved in cutaneous inflammation and autoimmune melanocyte damage.

## 2 Methodology

This narrative review was based on a structured literature search focused on the relationship between gut and skin microbiome alterations, microbial metabolites, immune dysregulation, oxidative stress, and melanocyte dysfunction in vitiligo. The literature search was conducted using indexed databases, including MEDLINE/PubMed, Scopus, Web of Science, and ScienceDirect. Recent literature was prioritized, particularly studies published within the last 10 years; however, earlier studies were included when they provided foundational evidence or essential mechanistic information relevant to the pathways discussed. Original human studies, experimental and animal studies, microbiome and metabolomic investigations, systematic reviews, meta-analyses, and narrative reviews were considered when directly relevant to the scope of this review. Search terms were selected according to the main objectives of the manuscript and included combinations of “vitiligo”, “gut microbiome”, “gut microbiota”, “skin microbiome”, “skin microbiota”, “dysbiosis”, “gut–skin axis”, “gut-skin metabolome”, “microbial metabolites”, “short-chain fatty acids”, “oxidative stress”, “reactive oxygen species”, “melanocyte dysfunction”, “immune dysregulation”, “T regulatory cells”, and “inflammasome”. Studies were selected according to their relevance to microbiome composition, microbial or host metabolic alterations, immune signaling, oxidative stress, and mechanisms potentially linking gut or skin dysbiosis to melanocyte damage and vitiligo progression. Evidence derived directly from patients with vitiligo was prioritized, while experimental models and mechanistic studies from related biological contexts were used to support pathway-level interpretation when direct evidence in vitiligo was limited.

## 3 Current microbiome and metabolome evidence in vitiligo

### 3.1 Gut microbiome alterations in vitiligo

Recently, the role of the gut microbiome alterations in vitiligo pathogenesis has been proposed (11). For instance, an abnormal reactive oxygen species accumulation in skin and gut dysbiosis were associated in a Vitiligo mice model. In this case, gut dysbiosis was related to an increased intestinal permeability, which allowed the translocation and accumulation of hippuric acid in the skin (10). This accumulation of hippuric acid was associated with the overexpression of genes involved in the production of reactive oxygen species, which triggered endoplasmic reticulum stress, DNA and cellular structures disruption, ultimately leading to melanocyte apoptosis and programmed cell death (10, 12–14). Moreover, in vitiligo mouse model treated with antibiotics there was a modification of gut microbiota abundance and composition, which promoted the upregulation of reactive oxygen species-related genes such as *Abcd2*, *Car3*, and *Aimf2*, oxidative stress-related genes such as *Lep* and *Mtarc1*, and immune-related genes such as *Tshr* and *Lipe*. Therefore, the role of gut microbiota in regulating extraintestinal organ-skin interactions, particularly in oxidative stress, immune response, and ROS accumulation in vitiligo disease states (10).

In a case-control study, there were significant differences (*P* < 0.05) in the alpha and beta diversity in the gut microbial composition between vitiligo cases versus control individuals. These vitiligo individuals showed an increased alpha diversity as well as lower *Bacteroidetes: Firmicutes* ratio (1.6: 1) versus healthy controls (2.3: 1). Moreover, it was observed that the abundance of *Negativicutes* was higher while the abundance of *Bacteroidia* was lower in patients with vitiligo. Notably, the abundance of *Negativicutes* gradually decreased with longer disease duration, whereas the abundance of the other bacterial classes remained largely unchanged. This study also detected several bacterial genera that were significantly different between vitiligo patients and the control group (*Subdoligranulum, Escherichia/Shigella, Ruminococcus 2, Gemmobacter*), which could be used as biomarkers to distinguish between them. Lastly, the presence of several microorganisms like *Corynebacterium 1, Ruminococcus 2, Jeotgalibaca*, and *Psychrobacter* were associated with the disease duration, serum IL-1β amounts, as well as its correlation with metabolites like taurine, uridine, nicotinamide, among others, in vitiligo individuals (15) Therefore, the characterization of the metabolites in the gut-plasma-skin axis could help with the identification of overproduced metabolites which could be involved in skin immunological overresponse and vitiligo pathogenesis.

Another study performed in Indian vitiligo patients also showed gut microbiome alterations, which was characterized by a different microbial community abundance, with no differences in alpha-and beta-diversity between controls and vitiligo individuals, and a significantly lower Firmicutes/Bacteroidota ratio (*P* = 0.005). At the taxonomic level, vitiligo samples showed increased abundance of genus like *Prevotella*, particularly *Prevotella copri*, and *Parabacteroides merdae*, whereas *Anaerovoracaceae, Enterobacteriaceae, Ruminococcaceae, Nanosynbacteraceae, Exiguobacteraaceae, Peptostreptococcaceae, Pseudomonadaceae, Gemellaceae*, and *Lachnospiraceae* were found to be depleted in the vitiligo group. The functional prediction performed in this study further suggested enrichment of microbial genes involved in mucin and glycan degradation, including hexosaminidase, alpha-L-fucosidase, sialate O-acetylesterase, and L-fucose transport, as well as pathways related to lipid metabolism, short-chain fatty acid biosynthesis, lipopolysaccharide biosynthesis, and lipid transport. These findings indicate that vitiligo-associated dysbiosis may involve impaired mucosal barrier integrity and altered microbial metabolic activity, providing a potential link between gut microbial imbalance and systemic immune dysregulation (11).

### 3.2 Skin microbiome dysbiosis

Another study described that patients with vitiligo showed reduced microbial α-diversity and an increased Firmicutes/Bacteroidetes ratio compared with healthy controls in the gut (*P* = 0.005), while an increased *Firmicutes*-to-*Bacteroidetes* ratio in patients with vitiligo was observed. In the skin, microbial alterations were more pronounced in lesional than in non-lesional sites, particularly in deep biopsies, where lesional skin showed distinct β-diversity, depleted commensal taxa such as *Bifidobacterium*, *Bacteroides*, *Parabacteroides*, *Enterococcus*, and *Escherichia/Shigella*, and enrichment of opportunistic or inflammation-associated bacteria, including *Mycoplasma*, *Streptococcus*, *Gemella*, and *Proteobacteria*-related genera. Lesional biopsies were also characterized by increased mitochondrial DNA signatures, which correlated with higher systemic IFN-γ, CXCL9, and CXCL16 levels, supporting a link between local microbial imbalance, tissue stress, and immune activation in vitiligo (16).

The dysbiosis in vitiligo with specific patterns of distribution across the skin and gut has also been described in a study of Ganju et al. (2016). In this study, paired cutaneous samples of lesional and non-lesional skin showed a dominant phylum structure composed mainly of *Actinobacteria*, *Proteobacteria*, *Firmicutes*, and *Bacteroidetes*. However, *Methylobacterium* constitutes the core genus exclusive to lesional skin, while *Anaerococcus*, *Microbacterium*, *Streptophyta* and *Nocardiodes* constitute core genera exclusive to non-lesional skin. Non-lesional skin showed higher abundance of *Corynebacterium*, whereas lesional skin was enriched in *Methylobacterium*, *Enhydrobacter*, *Paracoccus*, and *Staphylococcus*, suggesting localized remodeling of the cutaneous microbial ecosystem. Lastly, there was a greater diversity and higher association between microbial communities in the non-lesioned skin. Therefore, these findings suggest that vitiligo involves both local cutaneous dysbiosis at lesional skin, which could have a determinant role in the maintenance and severity of disease (17).

### 3.3 Metabolomic alterations in vitiligo

A microbiome–metabolome study suggests that gut dysbiosis in vitiligo is accompanied by alterations in fecal metabolic profiles and pathways involved in amino acid and vitamins metabolism. Patients with vitiligo showed reduced microbial α-diversity and distinct taxonomic composition, with an increased *Firmicutes*-*Bacteroidota* ratio, and augmented abundance of genera such as *Lachnoclostridium*, *Faecalibacterium*, *Staphylococcus*, *Megasphaera*, and *Anaerostipes*, while *Bilophila* and *Collinsella* showed lower levels of abundance. These microbial changes were associated with altered fecal metabolites, including increased oleamide, stearamide, creatine, taurine, and cuminaldehyde, while nicotinic acid, L-glutamic acid, indole, dl-stachydrine, and secnidazole were decreased. Correlation analyses further linked *Lachnoclostridium* negatively with indole and L-glutamic acid, whereas *Bilophila* was positively associated with these metabolites and negatively correlated with taurine, which suggested a potential disruption of amino acid, indole, bile acid, and taurine metabolism. In this study also analyzed the implications of metabolic dysregulation in several pathways, which resulted in a metabolic remodeling, identifying significant alterations in phenylalanine, tyrosine, and tryptophan biosynthesis, GABAergic synapse pathways, and taurine and hypotaurine metabolism. Together, these findings indicate that vitiligo-related dysbiosis may involve coordinated shifts in microbial ecology and metabolite production affecting immune and neuroactive metabolic pathways (18).

### 3.4 Integrated microbiome–metabolome studies

These studies have shown the role of gut microbiota in vitiligo pathogenesis, due to the metabolic products that could reach skin tissue and trigger inflammatory and autoimmune pathways. As described previously, gut microbiome is in charge of short-chain fatty acids biosynthesis, and gut dysbiosis can alter the production of key metabolites like butyrate, which has an important role as histone deacetylase inhibitor that subsequently disrupts the gene expression of immune cells (19). Moreover, gut dysbiosis and altered metabolites abundance can influence the expression level of Toll-like receptors (TLRs) of antigen presenting cells, contribute to Th17/Treg imbalance, and aberrant immune responses via epigenetic modifications, which could lead to autoimmune diseases like vitiligo (20). Moreover, the enrichment of *Lachnoclostridium* has been associated with CD8+T cells infiltration and overexpression of chemokines such as CXCL9/10 in vitiligo individuals (18). In addition, *Roseburia* is also elevated in vitiligo individuals and is also involved in CD8+T cell activation by its binding to the Toll-like receptor 5 (TLR5), which could potentially exacerbate autoimmune responses (18, 21), as is observed in vitiligo individuals. Furthermore, riboflavin metabolism is degraded in vitiligo individuals, that trigger mitochondrial dysfunction by defects of precursors for flavin cofactors, which are involved in mitochondrial electron transport and fatty acid β-oxidation (16, 22).

Overall, current evidence suggests that vitiligo is associated with alterations in the gut–skin axis microbiota and metabolome, involving altered bacterial homeostasis, SCFA biosynthesis, bacteria that trigger inflammation, and disruption of amino acid, indole, bile acid, taurine, and ROS producing metabolic pathways. These changes may contribute to an impaired gut barrier function, systemic immune activation, ROS accumulation, and melanocyte damage. Although causality remains unspecified, integrated gut microbiota and metabolome analysis could support a link between dysbiosis, metabolic remodeling, and autoimmune melanocyte loss. This evidence highlights the need for longitudinal and functional studies to define the role of gut microbiota and its metabolites in vitiligo onset, progression, and therapeutic approaches (**Table 1**).

**Table 1:** Chronological overview of gut and skin microbiome studies in vitiligo.

Year	Population/sample type	Methodology	Microbial alterations	Metabolites changes	Main findings	Ref.
2016	Patients with vitiligo/Lesional and non-lesional skin	16S rRNA gene sequencing and microbial community analysis	Decreased taxonomic richness, enrichment of *Methylobacterium*, *Enhydrobacter*, *Paracoccus*, and *Staphylococcus* in lesional skin; Increased of *Corynebacterium* in non-lesional skin	Not assessed	Localized cutaneous dysbiosis in vitiligo lesional skin.	(17)
2020	FH-A2D vitiligo-prone mice/Feces and skin	Oral antibiotic administration in mice pregnancy and pups of 1 g/L of ampicillin and 1 g/L of neomycin sulfate, and control mice; 16S rRNA gene sequencing and microbial community analysis; immune-cell profiling	Ampicillin: marked gut dysbiosis, decreased diversity and enrichment of *Pseudomonas*; Neomycin: *Bacteroides*-dominated gut community; Limited skin microbiome changes	Not assessed	Accelerated depigmentation by ampicillin, altered T-cell distribution and increased skin Tregs by neomycin. Functional evidence that gut microbiota perturbation can modify vitiligo progression.	(23)
2020	Individuals with vitiligo exposed to narrowband UV (NBUVB) light treatment/Skin swab of Lesional and non-lesional sites	16S rRNA gene sequencing and microbial community analysis	No alpha diversity differences between normal skin samples before NBUVB and lesional skin samples after NBUVB. Enrichment of *Pseudomonas*, *Propionibacterium* and *Lactobacillus* in lesional skin samples after NBUVB compared with normal skin samples before NBUVB, whereas *Staphylococcus*, *Bacillus* and *Psychrobacter* were enriched in normal skin samples before NBUVB compared with in lesional skin samples after NBUVB.	Not assessed	Decreased diversity from NBUVB irradiation lesional sites to lesional skin. No significant differences between normal skin and NBUVB irradiation lesional skin. NBUVB irradiation is helpful to maintaining the cutaneous microbiota balance for vitiligo patients.	(24)
2020	30 patients with advanced non-segmental vitiligo and 30 healthy controls/Feces and serum	16S rRNA gene sequencing and untargeted Liquid Chromatography-Mass Spectrometry (LC-MS) serum metabolomics	Increased alpha diversity, decreased *Bacteroidetes: Firmicutes* ratio. Vitiligo disease-associated bacteria: *Corynebacterium 1*, *Ruminococcus 2*, *Jeotgalibaca*, and *Psychrobacter*	Different levels of 23 serum metabolites in vitiligo individuals compared to controls, including: taurochenodeoxycholate, L-NG-monomethyl-arginine, taurine, uridine, nicotinamide, etc.	Several metabolites might be essential molecules for critical species in vitiligo patients’ microbiome. Coordinated alterations of gut microbiome and circulating metabolome associated with disease duration and IL-1β serum levels.	(15)
2021	20 subjects with vitiligo and healthy controls/Feces, serum, skin swabs of and biopsies of lesional and non-lesional sites	16S rRNA gene sequencing of superficial and deep skin and gut microbiota	Vitiligo patients showed decreased microbial richness in gut and deep lesional skin, increased phylogenetic diversity, depletion of *Bifidobacterium* and enrichment of Tenericutes, *Streptococcus* and *Mycoplasma* in deep lesional skin.	No metabolomics. Increased mitochondrial DNA signatures associated with innate immunity and stress marker.	Dysbiosis in gut and deep lesional skin of vitiligo individuals is linked with profound mitochondrial damage, loss of protective bacteria, and heightened innate immune response in the blood.	(16)
2023	25 patients with advanced non-segmental vitiligo and 25 matched healthy controls/feces	Shotgun metagenomic sequencing	Vitiligo patients showed a reduced α-diversity, increased relative abundance of *Bacteroides fragilis*, and decreased abundance of *Staphylococcus thermophiles*	Gut metabolic modules analysis showed downregulated cysteine degradation, and upregulated galactose degradation in patients with vitiligo.	Taxonomic gut microbiomes and metabolic pathways different in vitiligo individuals compared to heathy controls.	(25)
2023	32 patients with non-segmental vitiligo and 27 healthy controls/feces and serum	16S rRNA gene sequencing and metabolomic profiling by non-targeted liquid chromatography mass spectrometry	Differential gut microbial composition between vitiligo individuals and healthy controls. Increased *Firmicutes: Bacteroidetes*ratio, increased *Megamonas*, *Bifidobacterium*, *Psychrobacter*.	Paraxanthine, caffeine, xanthosine from caffeine methabolism are down-regulated in vitiligo individuals.	Gut microbial and metabolic alterations coexist in young adults with vitiligo.	(26)
2024	22 adults with vitiligo and 10 matched healthy controls/Feces	16S rRNA V3 gene sequencing; PICRUSt2 functional prediction.	There were no differences in alpha diversity between groups. Decreased *Firmicutes*/*Bacteroidota* ratio. Increased relative abundance of *Prevotella*, *Parabacteroides merdae*, and depletion of several commensal/SCFA-associated taxa	Functional predicted analysis revealed a higher abundance of fatty acid, lipid metabolism-related genes and mucin/glycan degradation in the vitiligo group.	Vitiligo-specific dysbiosis potentially associated with impaired mucosal barrier function and altered microbial metabolic capacity.	(11)
2024	Vitiligo-prone mice model receiving topical Neosporin and bacitracin/Feces and skin swabs	16S rRNA gene sequencing Topical antibiotic intervention.	Neosporin is associated with reduced abundance of *Alistipes* in the gut and reduced CD8+ T-cell abundance in the skin.	Not assessed	Neosporin can mediate skin Treg infiltration to limit vitiligo development, providing interventional evidence connecting microbiota modulation with cutaneous immunity.	(27)
2025	49 patients with vitiligo and 49 healthy controls/Feces	16S rRNA V3–V4 gene sequencing.	Decreased microbial diversity, lower Firmicutes/Bacteroidota ratio, and *Bacteroides* and *Parabacteroides* as disease-specific microbial marker genera	Not assessed	*Bacteroides* and *Parabacteroides* correlated positively with disease duration, and improving early diagnosis and developing potential treatment for vitiligo individuals.	(28)
2025	10 patients with vitiligo and 37 healthy controls/Feces	Whole-genome shotgun sequencing and Kyoto Encyclopedia of Genes and Genomes pathways for prediction analysis.	Reduced abundasnce of *Faecalibacterium prausnitzii*, *F. duncaniae*, and *Megamonas funiformis* and increased *Bifidobacterium bifidum*.	Significant enrichment in O-glycan biosynthesis pathways in vitiligo.	Active vitiligo showed reduced microbial diversity, greater interindividual variability, and altered predicted metabolic functions.	(19)
2025	28 vitiligo patients with depression/anxiety, 44 without emotional distress, and 37 healthy controls/Feces	16S rRNA sequencing and targeted LC–triple-quadrupole MS metabolomics.	Reduced α-diversity, *Lachnoclostridium* associated with vitiligo plus emotional distress; *Faecalibacterium* characterized vitiligo without emotional distress.	Variations in pathways involved in biosynthesis of phenylalanine, tyrosine, tryptophan, bile secretion, GABAergic synapses, taurine, hypotaurine metabolism between the vitiligo and healthy control.	Specific microbiome and metabolome remodeling may contribute to the pathogenesis of vitiligo, which could address neuropsychological comorbidity in vitiligo.	(18)
2025	15 vitiligo patients and 15 healthy controls, Experimental vitiligo C57BL/6 J mice model/Feces, serum and skin swabs	16S rRNA gene sequencing, gut microbiota depletion using antibiotics on vitiligo mice model (1 g/L ampicillin, 1 g/L metronidazole, 1 g/L neomycin, and 0.5 g/L vancomycin), metabolomics, and functional molecular assays.	High expression of oxidative stress-related genes in vitiligo lesions. Depletion of gut microbiota reduced ROS accumulation and mitochondrial dysfunction.	Accumulation of hippuric acid dependent of gut microbiota, which promotes ROS accumulation by NOS2 and MAPK14 proteins.	Evidence of the role of gut dysbiosis in accumulation of hippuric acid and skin oxidative stress of vitiligo individuals.	(10)
**2025**	21 patients with vitiligo and 6 healthy controls/skin swabs	16S rRNA V1–V9 sequencing before/after topical steroids and phototherapy	Alpha and beta showed no significant changes in posttreatment. Increased relative abundance of *Porphyromonas* spp., *Bacteroides salyersiae*, *Enterobacter kobei*, *and Corynebacterium coylea*.	The homolactic fermentation and glycolysis pathways decreased in vitiligo patients.	Differences were observed in the microbiomes of vitiligo patients and healthy controls, which could indicate the potential involvement in vitiligo pathogenesis and treatment response.	(29)

## 4 Microbiota and metabolic pathways in vitiligo

### 4.1 AhR signaling and immune–melanocyte disruption

The aryl hydrocarbon receptor (AhR) is a nuclear transcription factor involved in cellular homeostasis, pigmentation, and cutaneous immune regulation (30). AhR can be activated by different types of ligands, including endogenous ligands generated by biological systems, exogenous ligands derived from the diet, and synthetic ligands such as toxic agents or drugs (30). In the canonical pathway, cytoplasmic AhR binds to its ligands and translocates to the nucleus, where it dimerizes with the aryl hydrocarbon receptor nuclear translocator (ARNT) to form the AhR/ARNT complex. This complex recognizes specific DNA sequences known as xenobiotic response elements (XREs), thereby activating the transcription of target genes involved in biological and toxicological responses (31). These include genes encoding xenobiotic-metabolizing enzymes, such as *CYP1A1, CYP1A2*, and *CYP1B1*, as well as genes involved in cell differentiation, cell-cycle regulation, and immune-response coordination (32, 33).

Several tryptophan-derived metabolites, including kynurenine (KYN), kynurenic acid (KYNA), and 6-formylindolo[3, 2-b]carbazole (FICZ), are considered AhR ligands (32). In stable non-segmental vitiligo, activation of the kynurenine pathway has been described, along with increased expression of kynurenine aminotransferase (KYAT). This metabolic alteration promotes excessive KYNA production, which can activate AhR in keratinocytes and induce overexpression of the chemokine CXCL10. Consequently, dysregulation of this pathway may promote the expression of inflammation-related genes in lesional skin and contribute to the persistence of autoimmune inflammation in vitiligo (34). In addition, a significant reduction in AhR expression has been reported in lesional skin tissue, which may impair local homeostasis and immune regulation, thereby weakening protective mechanisms that normally help preserve melanocytes from autoimmune destruction (32).

Moreover, under homeostatic conditions, AhR regulates pathways such as Nrf2, PI3K/Akt, and Wnt/β-catenin, thereby connecting microbial tryptophan metabolites with antioxidant defense, melanocyte survival, and melanogenesis (2). Through the Nrf2 antioxidant pathway, the transcription of protective enzymes is stimulated, including heme oxygenase-1 (HO-1) and NAD(P)H quinone oxidoreductase 1 (NQO1) (35), AhR signaling may also activate the PI3K/Akt pathway, which helps inhibit oxidative stress-induced melanocyte apoptosis (36). In addition, AhR interacts with the Wnt/β-catenin pathway through the AKT/GSK-3β/β-catenin signaling cascade, promoting the activity of microphthalmia-associated transcription factor (MITF), a master regulator of melanogenic enzymes involved in melanin synthesis, including tyrosinase, TRP-1, and TRP-2 (2, 37). Conversely, disruption of these pathways may weaken melanocyte-protective mechanisms, impair melanin synthesis, and contribute to the persistence of inflammatory damage in vitiligo lesional skin (2).

### 4.2 SCFA-mediated GPCR/HDAC signaling and immune tolerance

Short-chain fatty acids (SCFAs), including acetate, propionate, and butyrate, are the main metabolites produced by microbial fermentation of non-digestible carbohydrates, such as dietary fiber, in the gut. These metabolites act as molecular messengers in several processes related to immune homeostasis in tissues such as the skin (38). SCFAs mediate immunomodulatory effects through the activation of G protein-coupled receptors (GPCRs) and the inhibition of histone deacetylases (HDACs) (39, 40).

These receptors include GPR41, GPR43, and GPR109A, which are expressed in epithelial and immune cells and are recognized as free fatty acid receptors (40, 41). Specifically, GPR41 shows greater affinity for propionate, GPR43 displays higher affinity for acetate and propionate, whereas GPR109A has greater affinity for butyrate (2, 40). In addition, butyrate and propionate promote HDAC inhibition, thereby modulating cellular gene expression and influencing inflammatory responses and immune cell differentiation, including B and T lymphocytes (42).

Furthermore, HDAC inhibition by butyrate and propionate increases histone acetylation, which promotes Foxp3 gene expression and enhances the suppressive function of regulatory T cells (Tregs). In the intestinal mucosa, this SCFA-induced process is an essential mechanism for maintaining immune tolerance and preventing excessive inflammatory responses (43).

Although much of the evidence comes from studies on intestinal epithelial and immune regulation, these mechanisms are relevant to vitiligo within the gut–skin axis, as SCFAs may be involved in immune balance, inflammation, and cutaneous immune responses (15, 16).

Consistent with this immunoregulatory role, patients with vitiligo have been reported to exhibit immune dysregulation characterized by reduced Foxp3 expression in peripheral blood as well as in lesional and perilesional skin. This reduced expression leads to functional and qualitative alterations in Tregs. In this context, the induction of Foxp3 acetylation through SCFA-mediated HDAC inhibition may represent a potential mechanism to restore cutaneous immune homeostasis (44).

### 4.3 IFN-γ–JAK/STAT–CXCL9/CXCL10 inflammatory amplification

The IFN-γ–JAK/STAT–CXCL9/CXCL10 axis is involved in inflammatory processes in vitiligo. This process begins when autoreactive CD8^+^ T cells recognize melanocyte antigens and start secreting interferon-gamma (IFN-γ). IFN-γ binds to its specific receptors, IFNGR1 and IFNGR2, on the surface of keratinocytes and dermal fibroblasts, triggering activation of the intracellular JAK/STAT signaling pathway (45).

This interaction activates JAK1 and JAK2, which phosphorylate STAT1. Once phosphorylated, STAT1 translocates to the nucleus, where it acts as a transcription factor and promotes the expression of the chemokines CXCL9 and CXCL10. These chemokines are then released into the cutaneous microenvironment, where they bind to CXCR3 on immune cells (46). CXCL9 primarily promotes the recruitment of additional CD8^+^ T cells from the bloodstream into the skin, whereas CXCL10 supports their localization and effector activity within skin lesions (47).

This process has been described in analyses of interstitial fluid from lesional skin, which show increased levels of IFN-γ-associated chemokines. In addition, patients with active vitiligo have higher plasma levels of IFN-γ and CXCL10, and serum concentrations of CXCL9/CXCL10 have been correlated with disease activity or severity (48).

Thus, inflammation is sustained because infiltrating immune cells continuously release cytokines such as IFN-γ, TNF-α, IL-2, and IL-15, maintaining local inflammation and promoting melanocyte damage. Moreover, memory T cells contribute to disease persistence even after periods of apparent clinical stability (49).

Furthermore, studies evaluating responses to JAK inhibitors suggest that clinical repigmentation and reduced immune activity may result from inhibition of the IFN-γ–mediated JAK/STAT signaling pathway (50).

### 4.4 NF-κB, inflammasome activation, and innate immune system

Besides melanocyte-directed adaptive immunity, vitiligo may involve an early stage of innate immune activation triggered by oxidative stress and tissue-derived danger signals (51). Stressed melanocytes can release damage-associated molecular patterns (DAMPs), including mitochondrial and endoplasmic reticulum components, nucleic acids, and stress-related proteins. These signals may activate keratinocytes, dendritic cells, macrophages, and other cells, generating a local inflammatory microenvironment that could promote the subsequent activation of adaptive autoimmune responses (51, 52).

NF-κB acts as a central transcriptional regulator that integrates innate signals derived from DAMPs, oxidative stress, and dysbiosis, thereby promoting a pro-inflammatory response (53). Its activation may induce the expression of pro-inflammatory cytokines, including IL-1β, IL-6, and TNF-α, together with chemokines such as CXCL9 and CXCL10 and adhesion molecules that promote the recruitment and activation of additional immune cells toward perilesional skin (52).

In addition, inflammasome activation, particularly involving the NLRP3 complex, has been proposed to occur through a two-step mechanism. The first step, known as priming, is triggered by the stimulation of Toll-like receptors (TLRs) and activates the NF-κB pathway, increasing the expression of NLRP3 and pro-IL-1β. The second step, known as activation, is driven by several processes, including oxidative stress, accumulation of reactive oxygen species (ROS), and cellular damage. These signals promote inflammasome assembly, caspase-1 activation, and the subsequent inflammatory response (54).

Although direct evidence in vitiligo-affected skin remains limited, genetic studies, animal models, and experimental data suggest that the NLRP3 and NLRP1 inflammasomes may contribute to vitiligo pathogenesis. In particular, oxidative stress can activate NLRP3 in keratinocytes, favoring cell recruitment and cutaneous T-cell responses (55).

Additionally, other cell types, such as keratinocytes and dermal fibroblasts, may contribute to disease progression. Keratinocytes promote melanocyte loss of adhesion and T-cell recruitment through the production of MMP-9, the chemokines CXCL9 and CXCL10, and the cytokine IL-15 (56), whereas dermal fibroblasts modulate local immune-cell accumulation in response to IFN-γ (57). Collectively, these cells may contribute to the persistence of the autoimmune response (52, 57).

## 5 Integrated molecular pathways from dysbiosis to melanocyte dysfunction

Alterations in the gut microbiota and their associated molecular mechanisms suggest that these changes may contribute to vitiligo through the gut–skin–immune axis (1). Human studies have identified alterations in microbial diversity and composition in the gut and lesional skin, together with changes in circulating or fecal metabolites and inflammatory markers (15, 16). These alterations may modify the availability of immunoregulatory metabolites, including short-chain fatty acids, bile acid derivatives, and tryptophan–indole metabolites, with potential consequences for epithelial barrier function, systemic inflammation, Treg-mediated tolerance, and Th1/Th17 balance (2). Analyses of the gut and skin microbiota further indicate that intestinal and cutaneous dysbiosis may coexist with mitochondrial and immune alterations in individuals with vitiligo (16). In addition, experimental studies in vitiligo-prone mouse models suggest that microbiota perturbation may alter both the progression of depigmentation and the cutaneous immune microenvironment (23, 27). Gut microbiota-dependent accumulation of hippuric acid has also been linked to oxidative stress, mitochondrial dysfunction, and melanocyte injury (10). Taken together, these studies support the biological plausibility of a gut–skin–immune axis in vitiligo, although further studies are needed to determine whether these changes contribute directly to disease development in humans.

These microbial alterations may modify the availability of microbial metabolites that mediate host–microbiota communication and regulate key processes, including immune responses and other systemic functions (**Figure 1**) (58). In this context, AhR-mediated tryptophan–kynurenine signaling, short-chain fatty acid-dependent immune modulation through GPCR and HDAC activity, amplification of the IFN-γ–JAK/STAT–CXCL9/CXCL10 axis, and activation of NF-κB- and inflammasome-related innate immune pathways may represent interconnected mechanisms through which dysbiosis contributes to inflammation, loss of immune tolerance, and melanocyte dysfunction in vitiligo (51, 52). Therefore, microbial imbalance may enhance dendritic cell and cytotoxic T-cell activation, increasing IFN-γ production and downstream JAK/STAT-dependent CXCL9/CXCL10 signaling in the skin. This chemokine axis recruits and sustains autoreactive CD8+ T cells around melanocytes, thereby amplifying local inflammation and promoting progressive melanocyte destruction (2, 25).

**Figure 1:**
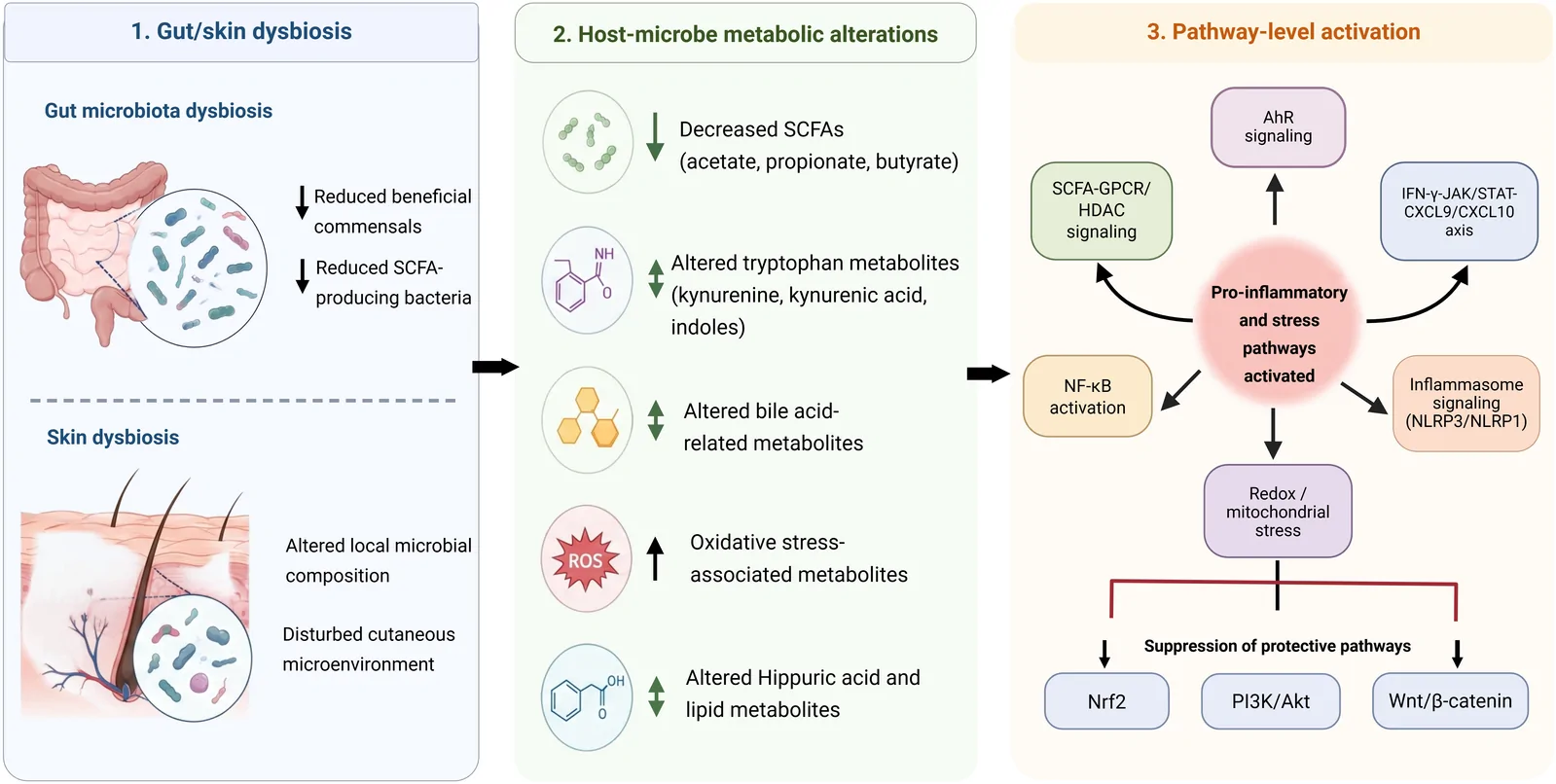
Gut-skin dysbiosis, host-microbe metabolic alterations, and pathway-level activation in vitiligo. Created in BIORENDER (https://BioRender.com/b4mrjpq). Gut and skin dysbiosis are associated with altered microbial composition and changes in SCFAs, tryptophan- and bile acid-related metabolites, hippuric acid, lipid metabolites, and oxidative stress-associated metabolites. These alterations may affect AhR, SCFA–GPCR/HDAC, IFN-γ–JAK/STAT–CXCL9/CXCL10, NF-κB/inflammasome, and redox–mitochondrial stress pathways, while impairing protective signaling involving Nrf2, PI3K/Akt, and Wnt/β-catenin.

After this immune amplification, the evidence also suggests that oxidative stress is an early event in melanocyte damage, linking cellular stress to persistent immune activation. At the cellular level, melanocytes exposed to oxidative stress exhibit mitochondrial dysfunction characterized by loss of membrane potential, impaired oxidative phosphorylation, and increased production of reactive oxygen species (2, 59). These events could reinforce CXCR3+ and CD8+ T-cell recruitment through the IFN-γ–CXCL9/CXCL10 axis and support the persistence of melanocyte-specific resident memory T cells in the skin, thereby sustaining chronic depigmentation and relapse (2, 52, 60). Therefore, dysbiosis-associated metabolic alterations could weaken immunoregulatory mechanisms, amplify inflammation, increase melanocyte susceptibility to mitochondrial dysfunction, impaired melanogenesis, apoptosis, and autoantigen exposure (61).

This process of metabolic stress, cellular damage, and autoantigen release may promote the persistence of tissue-resident memory CD8+ T cells in the epidermis, thereby sustaining autoreactive immune responses and contributing to the recurrence of depigmented lesions (59). Nevertheless, further studies are required to establish the direct contribution of the microbiota to these processes and to define more precisely its interactions with the immune responses involved in vitiligo.

## 6 Future perspectives

Future research should assess the role of microbiome–metabolome profiling toward mechanistic and longitudinal validation of the gut–skin axis in vitiligo. Moreover, multiethnic cohorts with standardized sampling of gut microbiota, lesional and non-lesional skin microbiota, serum metabolites, dietary variables, disease activity, and clinical outcomes are required to define reproducible microbial and metabolic signatures. Longitudinal studies should determine whether dysbiosis precedes disease onset, predicts progression, or reflects immune-mediated tissue damage. Mechanistic validation should prioritize pathways with direct biological plausibility, including tryptophan–kynurenine–AhR signaling, SCFA-mediated GPCR/HDAC regulation, IFN-γ–JAK/STAT–CXCL9/CXCL10 amplification, and NF-κB/inflammasome activation. Functional studies using melanocyte, keratinocyte, immune-cell, organoid, and animal models are needed to test whether specific microbial metabolites modulate oxidative stress, melanogenesis, Treg function, CD8+ T-cell recruitment, and resident memory T-cell persistence.

Regarding potential therapeutic strategies, experimental studies suggest that modulation of the gut or skin microbiota may influence depigmentation and local immune responses (2). However, evidence from clinical studies remains limited. Therefore, approaches such as dietary interventions, prebiotics, probiotics, fecal microbiota modulation, and metabolite-targeted strategies should still be considered experimental and require further clinical evaluation (62). More controlled clinical studies are needed to determine whether these approaches could complement established vitiligo therapies (**Figure 2**).

**Figure 2:**
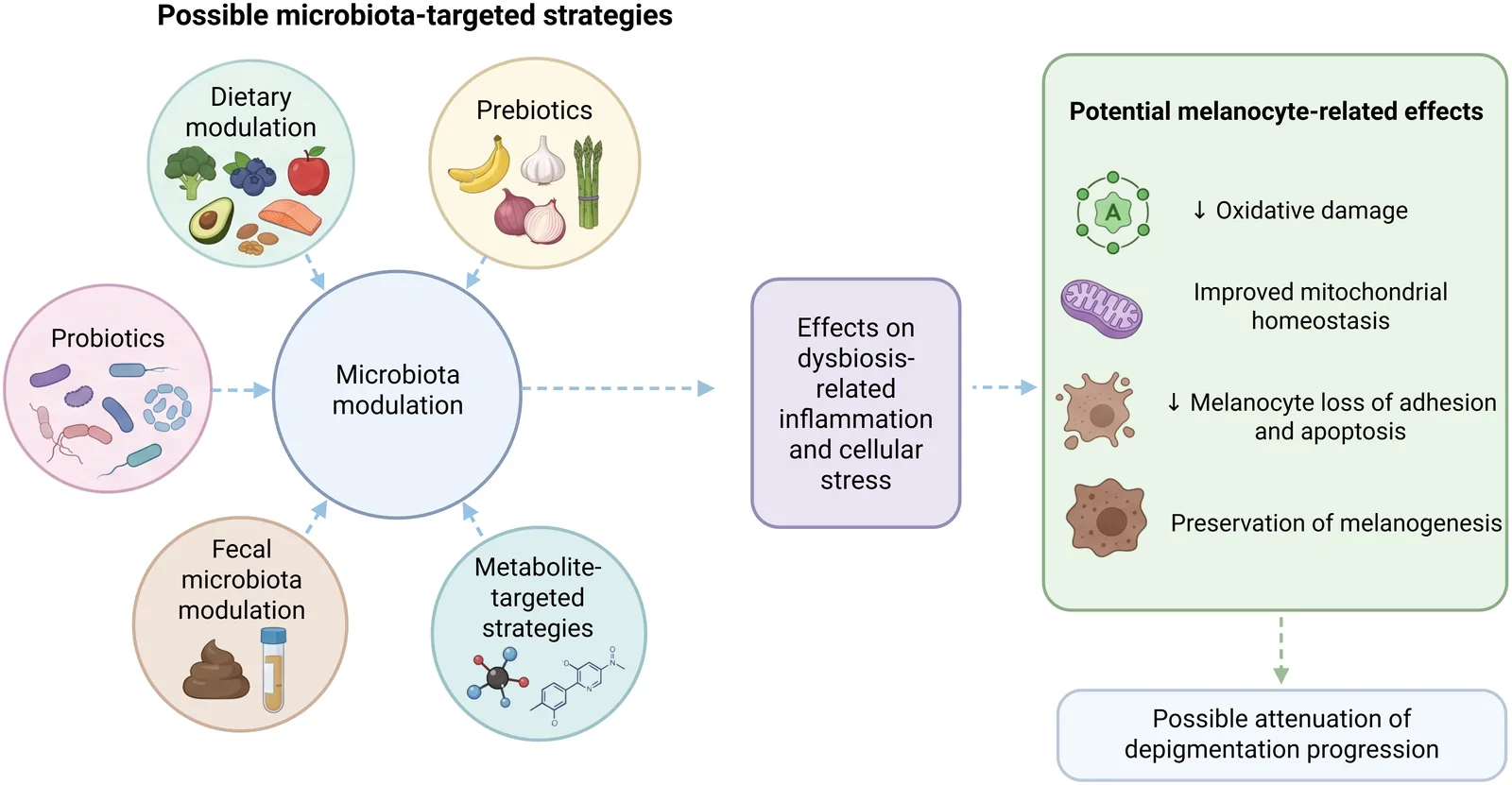
Potential microbiota-targeted interventions and downstream melanocyte-related effects in vitiligo. Possible microbiota-targeted strategies may influence dysbiosis-related inflammation and cellular stress, with potential effects on melanocyte oxidative damage, mitochondrial homeostasis, cell adhesion, apoptosis, and melanogenesis. These effects could contribute to the attenuation of depigmentation progression; however, clinical evidence remains limited. Created in BIORENDER (https://BioRender.com/srxobpe).

In this context, microbiome and metabolome findings may also help identify candidate biomarkers for disease monitoring and potential targets for adjunctive therapies.

## 7 Conclusion

Current evidence supports vitiligo as an immunometabolic disease in which gut and skin dysbiosis could contribute to melanocyte loss through altered microbial, metabolic, oxidative, and immune pathways. Altered microbial diversity and metabolite production may impair epithelial barrier function, reduce immune tolerance, promote systemic inflammation, and increase melanocyte susceptibility to mitochondrial dysfunction, ROS accumulation, impaired melanogenesis, apoptosis, and autoantigen exposure. At the pathway level, dysregulated AhR signaling, reduced SCFA-mediated immune regulation, IFN-γ–JAK/STAT–CXCL9/CXCL10 activation, and NF-κB/inflammasome priming provide a biologically plausible framework linking dysbiosis to autoimmune persistence. However, a direct causality has not been established. Therefore, microbial and metabolic signatures should be interpreted as candidate biomarkers and mechanistic hypotheses rather than validated drivers of vitiligo. Future longitudinal, multi-omics, and functional studies are needed to determine whether microbiome–metabolome modulation can prevent progression, reduce relapse, or improve clinical treatment response.

## References

[1] Nigro A Osman A Suryadevara P Cices A . Vitiligo and the microbiome of the gut and skin: A systematic review. Arch Dermatol Res 2024 317:1. (2025) 317:201–. doi: 10.1007/S00403-024-03679-6 39777551

[2] Yuan C Liu L Zeng D Yuan J Guo L Zhang J . Exploring the influence of gut microbiota metabolites on vitiligo through the gut-skin axis. Front Microbiol. (2025) 16:1566267. doi: 10.3389/FMICB.2025.1566267/FULL PMC12283607 40703229

[3] Akl J Lee S Ju HJ Parisi R Kim JY Jeon JJ . Estimating the burden of vitiligo: A systematic review and modelling study. Lancet Public Health. (2024) 9:e386–96. doi: 10.1016/S2468-2667(24)00026-4 38552651

[4] Seneschal J Bae JM Ezzedine K Hamzavi I Harris JE Bellei B . Vitiligo. Nat Rev Dis Primers. (2025) 11. doi: 10.1038/s41572-025-00670-x 41345471

[5] GLOVA Epidemiology Atlas GLOVA . Glova Epidemiology Atlas Glova. Available online at: https://www.globalvitiligoatlas.org/epidemiology-atlas (Accessed June 7, 2026).

[6] Roberts GHL Santorico SA Spritz RA . The genetic architecture of vitiligo. Pigment Cell Melanoma Res. (2020) 33:8–15. doi: 10.1111/pcmr.12848 31743585 PMC6928395

[7] Marchioro HZ Silva de Castro CC Fava VM Sakiyama PH Dellatorre G Miot HA . Update on the pathogenesis of vitiligo. Bras Dermatol. (2022) 97:478–90. doi: 10.1016/j.abd.2021.09.008 35643735 PMC9263675

[8] Chang WL Ko CH . The role of oxidative stress in vitiligo: An update on its pathogenesis and therapeutic implications. Cells. (2023) 12. doi: 10.3389/fmicb.2025.1566267 36980277 PMC10047323

[9] Liu X Liu J . Vitiligo: Are microbes to blame? Biosci Microbiota Food Health. (2025) 44:16–25. doi: 10.12938/BMFH.2024-051 39764486 PMC11700547

[10] Ni Q Xia L Huang Y Yuan X Gu W Chen Y . Gut microbiota dysbiosis orchestrates vitiligo-related oxidative stress through the metabolite hippuric acid. Microbiome. (2025) 13:112. doi: 10.1186/S40168-025-02102-0 40329424 PMC12054231

[11] Kumar S Mahajan S Kale D Chourasia N Khan A Asati D . Insights into the gut microbiome of vitiligo patients from India. BMC Microbiol 2024 24:1. (2024) 24:440. doi: 10.1186/S12866-024-03529-5 39468434 PMC11514916

[12] Chen J Li S Li C . Mechanisms of melanocyte death in vitiligo. Med Res Rev. (2021) 41:1138–66. doi: 10.1002/MED.21754 33200838 PMC7983894

[13] Chen J Wang Y Dai W Xu X Ni Q Yi X . Oxidative stress-induced hypermethylation and low expression of ANXA2R: Novel insights into the dysfunction of melanocytes in vitiligo. J Dermatol Sci. (2024) 114:115–23. doi: 10.1016/j.jdermsci.2024.02.009 38806323

[14] Yi X Guo W Shi Q Yang Y Zhang W Chen X . SIRT3-dependent mitochondrial dynamics remodeling contributes to oxidative stress-induced melanocyte degeneration in vitiligo. Theranostics. (2019) 9:1614–33. doi: 10.7150/THNO.30398 31037127 PMC6485185

[15] Ni Q Ye Z Wang Y Chen J Zhang W Ma C . Gut microbial dysbiosis and plasma metabolic profile in individuals with vitiligo. Front Microbiol. (2020) 11:592248. doi: 10.3389/fmicb.2020.592248 PMC7768019 33381090

[16] Bzioueche H Simonyté Sjödin K West CE Khemis A Rocchi S Passeron T . Analysis of matched skin and gut microbiome of patients with vitiligo reveals deep skin dysbiosis: Link with mitochondrial and immune changes. J Invest Dermatol. (2021) 141:2280–90. doi: 10.1016/J.JID.2021.01.036 33771527

[17] Ganju P Nagpal S Mohammed MH Nishal Kumar P Pandey R Natarajan VT . Microbial community profiling shows dysbiosis in the lesional skin of vitiligo subjects. Sci Rep 2016 6:1. (2016) 6:18761. doi: 10.1038/srep18761 26758568 PMC4725359

[18] Luan M Mao B Zhao Y Chen J Yang P Li W . Landscapes of gut microbiome and metabolic signatures in vitiligo patients with co-morbid emotional distress. J Dermatol Sci. (2025) 118:92–100. doi: 10.1016/J.JDERMSCI.2025.04.011 40368677

[19] Ju HJ Song WH Shin JH Lee JH Bae JM Lee YB . Characterization of gut microbiota in patients with active spreading vitiligo based on whole-genome shotgun sequencing. Int J Mol Sci 2025 Vol 26. (2025) 26. doi: 10.3390/IJMS26072939 40243573 PMC11988336

[20] Chen B Sun L Zhang X . Integration of microbiome and epigenome to decipher the pathogenesis of autoimmune diseases. J Autoimmun. (2017) 83:31–42. doi: 10.1016/J.JAUT.2017.03.009 28342734

[21] Kang X Liu C Ding Y Ni Y Ji F Lau HCH . Roseburia intestinalis generated butyrate boosts anti-PD-1 efficacy in colorectal cancer by activating cytotoxic CD8+ T cells. Gut. (2023) 72:2112. doi: 10.1136/GUTJNL-2023-330291 37491158 PMC10579466

[22] Henriques JB Olsen KR Bross P Gomes CM . Emerging roles for riboflavin in functional rescue of mitochondrial β-oxidation flavoenzymes. Curr Med Chem. (2010) 17:3842–54. doi: 10.2174/092986710793205462 20858216

[23] Dellacecca ER Cosgrove C Mukhatayev Z Akhtar S Engelhard VH Rademaker AW . Antibiotics drive microbial imbalance and vitiligo development in mice. J Invest Dermatol. (2019) 140:676. doi: 10.1016/J.JID.2019.08.435 31472106 PMC9851193

[24] Yuan X Wang L Meng D Wu L Wang X Zhang D . The impact of NBUVB on microbial community profiling in the lesional skin of vitiligo subjects. Microb Pathog. (2020) 140:103943. doi: 10.1016/J.MICPATH.2019.103943 31917273

[25] Luan M Niu M Yang P Han D Zhang Y Li W . Metagenomic sequencing reveals altered gut microbial compositions and gene functions in patients with non-segmental vitiligo. BMC Microbiol. (2023) 23:265. doi: 10.1186/S12866-023-03020-7 37737154 PMC10515041

[26] Wu Q Cheng P Shao T Li Z Ji Q Wang L . Alterations of gut microbiota and gut metabolites in the young-adult vitiligo patients. J Eur Acad Dermatol Venereology. (2023) 37:e904–7. doi: 10.1111/JDV.19012 36883792

[27] Touni AA Sohn R Cosgrove C Shivde RS Dellacecca ER Abdel-Aziz RTA . Topical antibiotics limit depigmentation in a mouse model of vitiligo. Pigment Cell Melanoma Res. (2024) 37:583–96. doi: 10.1111/PCMR.13164 38439216

[28] Dou Y Niu Y Shen H Wang L Lv Y Liu S . Identification of disease-specific gut microbial markers in vitiligo. Front Microbiol. (2025) 16:1499035. doi: 10.3389/fmicb.2025.1499035 PMC11833150 39967732

[29] Lai PT Lee CH Tseng HC . Dynamic changes of the skin microbiome in vitiligo patients after intervention with phototherapy and topical steroids: An observational study. Dermatologica Sin. (2025) 43:194–204. doi: 10.4103/DS.DS-D-24-00149

[30] Fernández-Gallego N Sánchez-Madrid F Cibrian D . Role of AHR ligands in skin homeostasis and cutaneous inflammation. Cells. (2021) 10:3176. doi: 10.3390/CELLS10113176 34831399 PMC8622815

[31] Yang J Qiao P Wang G Dang E . The role of aryl hydrocarbon receptor in skin homeostasis: Implications for therapeutic strategies in skin disorders. Cell Biochem Funct. (2025) 43. doi: 10.1002/CBF.70047 39866071

[32] Szelest M Walczak K Plech T . A new insight into the potential role of tryptophan-derived AhR ligands in skin physiological and pathological processes. Int J Mol Sci 2021 Vol 22. (2021) 22:1–31. doi: 10.3390/IJMS22031104 33499346 PMC7865493

[33] Coumoul X Barouki R Esser C Haarmann-Stemmann T Lawrence BP Lehmann J . The aryl hydrocarbon receptor: Structure, signaling, physiology and pathology. Signal Transduction Targeted Ther 2025 11:1. (2026) 11:20. doi: 10.1038/s41392-025-02500-8 41540003 PMC12808330

[34] Chen Z Li Y Tan X Nie S Chen B Mei X . Dysregulated tryptophan metabolism and AhR pathway contributed to CXCL10 upregulation in stable non-segmental vitiligo. J Dermatol Sci. (2024) 115:33–41. doi: 10.1016/j.jdermsci.2024.06.003 38955622

[35] Yang L Yang F Teng L Katayama I . 6-Shogaol protects human melanocytes against oxidative stress through activation of the Nrf2-antioxidant response element signaling pathway. Int J Mol Sci. (2020) 21. doi: 10.3390/IJMS21103537 32429495 PMC7279012

[36] Lu W Zhao Y Kong Y Zhang W Ma W Li W . Geniposide prevents H2 O2 -induced oxidative damage in melanocytes by activating the PI3K-Akt signalling pathway. Clin Exp Dermatol. (2018) 43:667–74. doi: 10.1111/CED.13409 29512905

[37] Zhao SJ Jia H Xu XL Bu WB Zhang Q Chen X . Identification of the role of Wnt/β-catenin pathway through integrated analyses and *in vivo* experiments in vitiligo. Clin Cosmet Investig Dermatol. (2021) 14:1089–103. doi: 10.2147/CCID.S319061 34511958 PMC8423189

[38] Morrison DJ Preston T . Formation of short chain fatty acids by the gut microbiota and their impact on human metabolism. Gut Microbes. (2016) 7:189. doi: 10.1080/19490976.2015.1134082 26963409 PMC4939913

[39] Schwarz A Bruhs A Schwarz T . The short-chain fatty acid sodium butyrate functions as a regulator of the skin immune system. J Invest Dermatol. (2017) 137:855–64. doi: 10.1016/j.jid.2016.11.014 27887954

[40] Du Y He C An Y Huang Y Zhang H Fu W . The role of short chain fatty acids in inflammation and body health. Int J Mol Sci. (2024) 25:7379. doi: 10.3390/IJMS25137379 39000498 PMC11242198

[41] Venegas DP De La Fuente MK Landskron G González MJ Quera R Dijkstra G . Short chain fatty acids (SCFAs)mediated gut epithelial and immune regulation and its relevance for inflammatory bowel diseases. Front Immunol. (2019) 10:424615. doi: 10.3389/fimmu.2019.00277 PMC6421268 30915065

[42] Xu Z Wang T Wang Y Li Y Sun Y Qiu HJ . Short-chain fatty acids: Key antiviral mediators of gut microbiota. Front Immunol. (2025) 16:1614879. doi: 10.3389/fimmu.2025.1614879 PMC12331605 40787446

[43] Sharma A Sharma G Im SH . Gut microbiota in regulatory T cell generation and function: Mechanisms and health implications. Gut Microbes. (2025) 17:2516702. doi: 10.1080/19490976.2025.2516702 40517372 PMC12169050

[44] Giri PS Mistry J Dwivedi M . Meta-analysis of alterations in regulatory T cells’ frequency and suppressive capacity in patients with vitiligo. J Immunol Res. (2022) 2022:6952299. doi: 10.1155/2022/6952299 36164321 PMC9508461

[45] Liu H Wang Y Le Q Tong J Wang H . The IFN-γ-CXCL9/CXCL10-CXCR3 axis in vitiligo: Pathological mechanism and treatment. Eur J Immunol. (2024) 54:2250281. doi: 10.1002/EJI.202250281 37937817

[46] Qiu Y Su X Chen J Jiang S Tang X He Y . Recent advances in vitiligo treatment. Immunotargets Ther. (2026) 15:1–11. doi: 10.2147/ITT.S577653 41940356 PMC13044860

[47] Speeckaert R Caelenberg Van E Belpaire A Speeckaert MM Geel van N . Vitiligo: From pathogenesis to treatment. J Clin Med. (2024) 13:5225. doi: 10.3390/JCM13175225 39274437 PMC11396398

[48] Chang Y Kang P Cui T Guo W Zhang W Du P . Pharmacological inhibition of demethylzeylasteral on JAK-STAT signaling ameliorates vitiligo. J Trans Med 2023 21:1. (2023) 21:434. doi: 10.1186/S12967-023-04293-2 37403086 PMC10318684

[49] Passeron T Seneschal J Picardo M Xiang L Tanemura A Winkler A . The role of JAK3 and TEC family kinases in vitiligo pathogenesis. Front Immunol. (2026) 17:1662245. doi: 10.3389/fimmu.2026.1662245 PMC13033657 41918735

[50] Qi F Liu F Gao L . Janus kinase inhibitors in the treatment of vitiligo: A review. Front Immunol. (2021) 12:790125. doi: 10.3389/fimmu.2021.790125 PMC8636851 34868078

[51] Pupo Silvestrini AV Lucas JM de Barros Cardoso CR Lopes Badra Bentley MV . Vitiligo as a failure of immune resolution and tissue regeneration: From stress signals to targeted immune modulation. Clin Rev Allergy Immunol. (2026) 69:42. doi: 10.1007/S12016-026-09165-3 42105025 PMC13157458

[52] Wang Y Cao J Liu X Xiong C Xu D Bian F . Innate immune activation in vitiligo: Mechanisms and pathophysiological implications. Front Immunol. (2025) 16:1631074. doi: 10.3389/fimmu.2025.1631074 PMC12568694 41169396

[53] Anilkumar S Wright-Jin E . NF-KB as an inducible regulator of inflammation in the central nervous system. Cells. (2024) 13. doi: 10.3390/CELLS13060485 38534329 PMC10968931

[54] Abais JM Xia M Zhang Y Boini KM Li PL . Redox regulation of NLRP3 inflammasomes: ROS as trigger or effector? Antioxid Redox Signal. (2015) 22:1111. doi: 10.1089/ARS.2014.5994 25330206 PMC4403231

[55] Hu G Ryter SW Meng G Zhou F Tang L . Inflammasomes in common immune-related skin diseases. Front Immunol. (2020) 11:882. doi: 10.3389/FIMMU.2020.00882 32528469 PMC7247819

[56] Touni AA Shivde RS Echuri H Abdel-Aziz RTA Abdel-Wahab H Kundu RV . Melanocyte-keratinocyte cross-talk in vitiligo. Front Med (Lausanne). (2023) 10:1176781. doi: 10.3389/fmed.2023.1176781 PMC10235633 37275386

[57] Xu Z Chen D Hu Y Jiang K Huang H Du Y . Anatomically distinct fibroblast subsets determine skin autoimmune patterns. Nature. (2021) 601:118–24. doi: 10.1038/s41586-021-04221-8 34912121

[58] Zeng L Qian Y Cui X Zhao J Ning Z Cha J . Immunomodulatory role of gut microbial metabolites: Mechanistic insights and therapeutic frontiers. Front Microbiol. (2025) 16:1675065. doi: 10.3389/fmicb.2025.1675065 PMC12484209 41040871

[59] Lv J Xu W Jiang P Yu W Xue H Hu N . Mitochondrial DNA release via VDAC1 in keratinocytes: A key driver of innate immunity and vitiligo pathogenesis. Cell Death Dis. (2026) 17:318. doi: 10.1038/s41419-026-08585-5 41851094 PMC13039960

[60] TurkMJ HuangYH . The immunology of vitiligo. Nat Rev Immunol. (2026) 26:284–97. doi: 10.1038/s41577-025-01249-z41482575

[61] Jimbow K Chen H Park JS Thomas PD . Increased sensitivity of melanocytes to oxidative stress and abnormal expression of tyrosinase-related protein in vitiligo. Br J Dermatol. (2001) 144:55–65. doi: 10.1046/J.1365-2133.2001.03952.X 11167683

[62] Al-Smadi K Leite-Silva VR Filho NA Lopes PS Mohammed Y . Innovative approaches for maintaining and enhancing skin health and managing skin diseases through microbiome-targeted strategies. Antibiotics. (2023) 12:1698. doi: 10.3390/ANTIBIOTICS12121698 38136732 PMC10741029

